# Primary breast lymphoma: Case report and literature review

**DOI:** 10.1016/j.radcr.2025.12.046

**Published:** 2026-01-17

**Authors:** Lana R.A. Pshtiwan, Ari M. Abdullah, Abdulwahid M. Salih, Dana O. Karim, Karzan M. Salih, Bayar B. Ameen, Sakar O. Arif, Masty Karim Ahmed, Sara Nasir Ahmed, Ali D. Saadullah, Fahmi H. Kakamad

**Affiliations:** a Scientific Affairs Department, Smart Health Tower, Madam Mitterrand Street, Sulaymaniyah, Iraq; b Department of Pathology, Sulaymaniyah Teaching Hospital, Sulaymaniyah, Iraq; c College of Medicine, University of Sulaimani, Madam Mitterrand Street, Sulaymaniyah, Iraq; d Kscien Organization for Scientific Research (Middle East Office), Hamid Street, Azadi Mall, Sulaymaniyah, Iraq

**Keywords:** Primary breast lymphoma, Extranodal non-Hodgkin lymphoma, Breast malignancy

## Abstract

Primary breast lymphoma (PBL) accounts for a small fraction of breast malignancies and is uncommon in young adults, yet it can lead to significant complications. This report describes a case of PBL in a young female patient. An 18-year-old female presented with a mass in the right breast with fever and sweating. Examination revealed a poorly defined right breast mass. Imaging showed a diffuse infiltrative process, and core biopsy with staining identified the lymphoid origin of the infiltrative disease. Chemotherapy was opted for, and follow-up for months has revealed no complications. Ten cases were reviewed. The mean age was (19.7). The most common symptom was a palpable breast mass(s) (60%). Imaging showed variable signal intensities of the breast masses. Immunohistochemistry and flow cytometry of tissue biopsies were the main methods of diagnosis. Large B-cell non-Hodgkin lymphoma was the most common diagnosis (60%). Chemotherapy was utilized in all of the cases with variations regarding regimens. Death and remission were seen in near equal proportions (40% vs 50%). Young women are not exempt from primary breast lymphoma, and chemotherapy might offer fruitful outcomes.

## Background

Primary breast lymphoma (PBL) is a malignancy that arises from the B-cells and T-cells of the breast lymphoid tissue, making it a non-Hodgkin lymphoma (NHL) [1]. Making up about 0.04%-0.74% of all breast malignancies, 0.7% of extra-nodal NHLs, and 1% of all NHLs makes this a rare condition [1]. Over 98% of cases are diagnosed in women [2]. The presenting age varies from teenagers to the elderly, with a younger mean age in East Asian countries such as China (45-47 years old) compared to the West (above 55-62 years old) [1]. The majority of PBL cases involve only 1 breast at the initial diagnosis, while bilateral PBL can be seen in 1%-14%, most of whom are pregnant or lactating [1].

B-cell lymphomas make up over 90% of PBLs, with >50% being Diffuse Large B-Cell Lymphoma (DLBCL), followed by other less common subtypes such as Follicular, Burkitt, and Marginal Zone lymphomas, while anaplastic large cell lymphoma (ALCL) makes up most of T-cell lymphomas [1,2]. Co-existence of more than 1 subtype of B-cell can happen, suggesting transformation from 1 to the other [2].

Estrogen is postulated to have a role in PBL due to the high prevalence of PBL in females, as well as the 29% increased risk of overall NHL in women receiving unopposed estrogen replacement therapy. Pre-existing autoimmune conditions may play a role in the development of breast lymphoma due to chronic inflammation, as is seen in other parts of the body [2]. Other postulated factors include pregnancy and lactation-related hormonal changes and breast implants. Microorganisms are not mentioned in literature as a potential factor for PBL, unlike their significant association with nodal and other extranodal non-Hodgkin lymphoma [1,2]

Pathological specimens with staining are crucial for diagnosis, as neither clinical nor imaging investigations are diagnostic. Combination chemotherapy is the treatment of choice, and central nervous system prophylaxis is being incorporated more into the regimen of earlier stages to ensure complete remission without relapse [2].

This report presents a case with an unusual age of presentation and the lack of common risk factors for PBL. The CaRel guideline has been followed throughout the presenting report, and non-peer-reviewed sources were excluded [3,4].

## Case presentation

### Patient information

An unmarried 18-year-old female presented with a 1-month history of a lumpy sensation in the right breast, accompanied by B-symptoms. She denied any local symptoms such as pain, skin discoloration, nipple discharge, nipple or skin changes, or swelling in other areas. Her systemic review was unremarkable. Her past surgical history included an eye surgery, while her past medical, drug, social, and gynecological histories were non-contributory. There was no family history of malignancy.

### Clinical examination

General examination revealed a comfortable-looking female without signs of respiratory distress or cachexia. Breast examination showed a grossly enlarged right breast with a single, palpable, large mass occupying the whole breast. The mass was large, irregular, poorly defined, and fixed to the underlying structures. The overlying skin appeared stretched but intact without identifiable ulcerations, discolorations, or peau d’orange. No lymphadenopathy nor any pathology elsewhere in the body was noted.

### Investigations

**Fig. 1:**
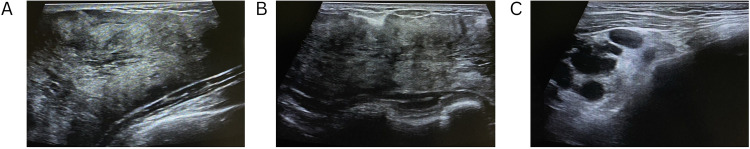
Grayscale ultrasonography of the breasts.

**Fig. 2:**
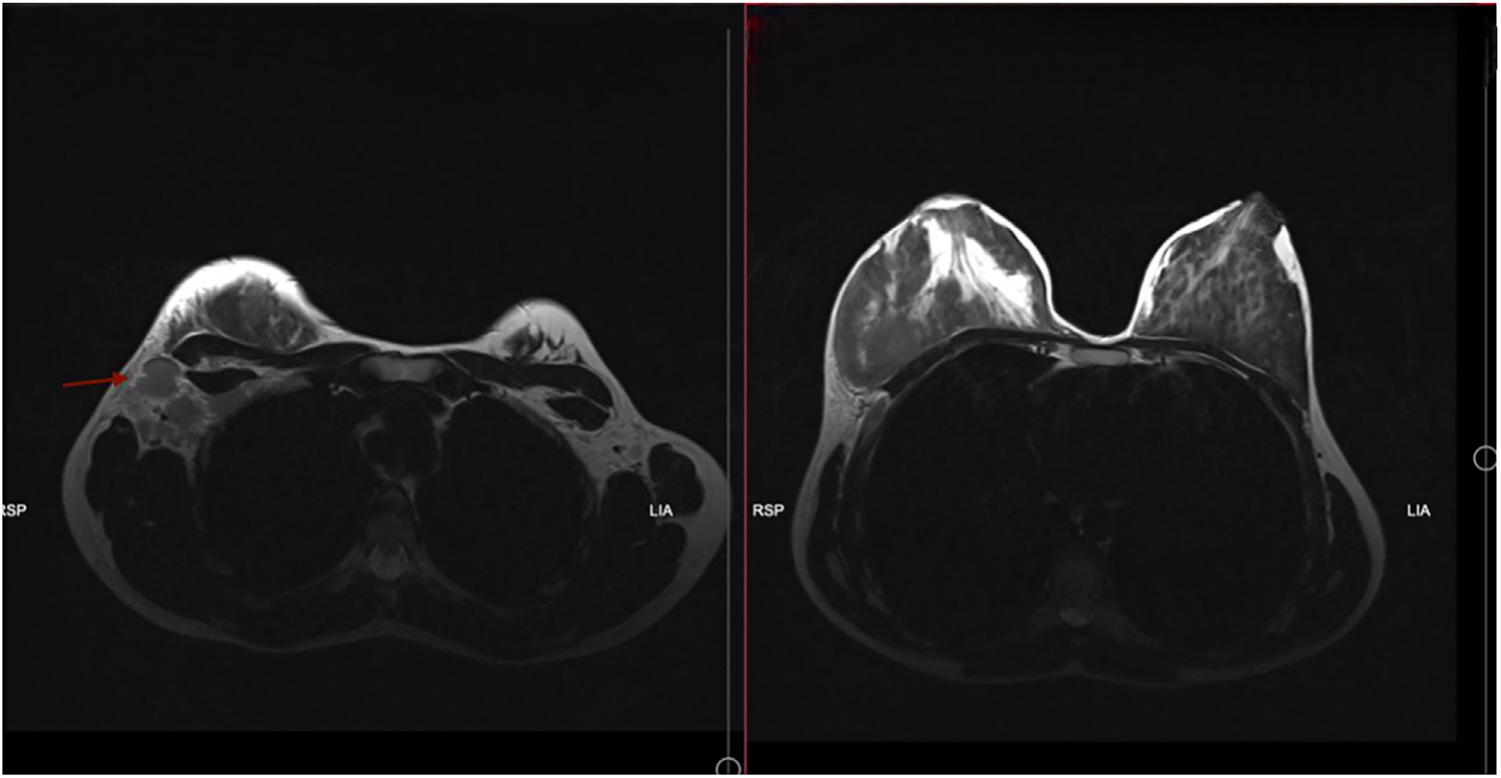
Axial view of T2 sequence of MRI of the chest.

Ultrasonography of the right breast revealed a diffusely heterogeneous echogenic glandular parenchyma seemingly displaced by an abnormal infiltrative tissue process without any discrete mass. Additionally, multiple matted and morphologically abnormal lymph nodes were observed involving all axillary levels as well as the supraclavicular region. The most prominent lymph node was located in axillary Level I, measuring 37 × 17 mm with a cortical thickness of 5.5 mm (Fig. 1). These findings were consistent with BI-RADS category 4/A4. Dynamic contrast-enhanced breast magnetic resonance imaging (MRI) demonstrated diffuse heterogeneous enhancement of the right breast parenchyma without a discrete mass or architectural distortion, accompanied by mild edema (Fig. 2). Diffusion-weighted imaging showed variable ADC signals with a Type I kinetic curve. Multiple large, matted lymph nodes were identified in all axillary levels, the largest measuring 22 × 20 mm. Additional abnormal nodes were seen in the supraclavicular and posterior cervical regions. The findings were classified as MR-4. MRI of the left breast shows a faint focal non-mass-like enhancement in the lateral central part measuring 14 × 12 mm and a 15 × 7 mm mass in the medial central part with a type I kinetic curve. Axilla: A few enlarged nodes were seen in the level I, measuring 13 × 8 mm A3. Contrast-enhanced CT of the neck, chest, and abdomen revealed enlargement of the right breast with heterogeneous density, suspicious for tumor infiltration, along with lymph node findings comparable to those seen on MRI. Core biopsy of the right breast mass revealed malignant lymphoid infiltration, consistent with secondary involvement of the breast by a lymphoid neoplasm. Fine-needle aspiration (FNA) of the right axillary lymph nodes demonstrated atypical lymphoid-like cells, suspicious for malignancy. Immunohistochemical (IHC) staining showed diffuse and strong cytoplasmic-membranous positivity for CD45, supporting the lymphoid origin of the tumor cells. The neoplastic cells were negative for estrogen receptor (ER), progesterone receptor (PR), and HER2 (score 0). The Ki-67 proliferation index was 65%.

### Intervention

A decision was made to give Rituximab with Cyclophosphamide, Doxorubicin, Vincristine, and prednisone (R-CHOP)

### Follow-up

Routine follow-up for 4 months has not shown any complications.

## Discussion

Patients with PBL at the time of diagnosis have a mean age in the 50s and 60s across many reviews [1,2]. The mean age of the reviewed cases was 19.7 (Table 1) [[5], [6], [7], [8], [9], [10], [11], [12], [13], [14]]. In the US, PBL has an incidence rate of 1.53-1.65/million persons of females [15]. The reported case is exceptional due to her age.

**Table 1:** Primary breast lymphoma reviewed cases.

First author (y)	Age (y)	Gender	Presentation	Clinical examination	U/S	MRI	HPE	Management	Outcome	Follow up (mo)
Daneshbod et al. (2010) [5]	16	Female	Breast pain, swelling, erythema, with heaviness	Breast inflammation with axillary and supraclavicular LAN	N/A	N/A	ALK+ve ALCL^d^	CHOP	Death	Few months
Charfi et al. (2020) [6]	16	Female	Breast erythema	Diffuse breast thickening and induration with variable edema	Increased echogenicity in breast parenchyma	N/A	ALK+ve ALCL	CTx^a^	CR	24
Restive et al. (2021) [7]	15	Female	Palpable breast mass	Two solid masses	One 6 cm mass and two smaller masses with hypervascularity	A 6.5 cm mass	DLBCL	CTx^b^	CR	20
Priyono and Effendy (2023) [12]	28	Female	Painful breast and axillary mass with fever	Necrotizing mass	Hypoechoic 5.9 cm mass in the left breast and 1.59 cm in the right breast with hypervascularity (BI-RADS 5)	N/A	High-grade BCNHL	R-CHOP	CR	N/A
El Khoury et al. (2021) [13]	25	Female	Palpable breast mass	Firm mass	Hypervascular and mixed echogenic masses	mildly hyperintense masses	BCL	R-CHOP	CR	3
Mremi et al. (2024) [14]	25	Female	Breast mass with arm pain	Palpable non-painful breast mass with ipsilateral axillary LAN	N/A	N/A	DLBCL	Prednisolone and allopurinol followed by R-CHOP	N/A	N/A
Hashemi et al (2009) [8]	17	Female	Bilateral painless breast mass	Bilateral irregular masses	Masses on both sides	N/A	Large BCL	CHOP with IV Dexamethasone	Death	N/A
Mungen et al. (2024) [9]	16	Female	Swelling in the breast	Tender breast swelling and induration	Hypoechoic, ill-defined masses with edema	N/A	Burkitt Lymphoma	CTx^c^	Death	3
Sathyanarayanan et al. (2014) [10]	19	Female	Breast mass	Firm breast mass	N/A	N/A	ALK+ve ALCL^d^	CHOP	CR	23
Lahlimi et al. (2025) [11]	20	Female	Bilateral breast nodules and enlargement	Right breast mass, enlargement, and nipple retraction, left breast mass	Left: fibroadenoma-like appearance, right: infectious mastitis	N/A	DLBCL	R-CHOP (4 cycles) then R-ICE	Death	4

AIEOP LNH-97: Italian association of pediatric hematology and oncology lymphoma protocol 1997, ALCL: anaplastic large cell lymphoma, ALK: anaplastic lymphoma kinase, BCL: B-cell lymphoma, BCNHL: B-cell non-Hodgkin lymphoma, CNs: central nervous system, CR: complete remission, CTx: chemotherapy, DLBCL: diffuse large B-cell lymphoma, EICNHL: European intergroup for childhood non-Hodgkin lymphoma

HPE: histopathologic examination, ITMTX: intrathecal methotrexate, LAN: lymphadenopathy, MACOP-B: methotrexate, doxorubicin, cyclophosphamide, vincristine, prednisone, and bleomycin, MRI: magnetic resonance imaging; N/A: not available, R-CHOP: rituximab, cyclophosphamide, doxorubicin (hydroxydaunorubicin), vincristine (oncovin), and prednisone, R-ICE: rituximab, ifosfamide, carboplatin, and etoposide, RTx: radiotherapy, U/S: ultrasound.

a CTx used here is doxorubicin, bleomycin, vincristine, and dacarbazin.

b AIEOP LNH-97 protocol plus rituximab.

c EICNHL group C3 protocol.

d The given dimensions on imaging are the largest or the only one provided.

Estrogen, as evident by more than 95% of patients being females, and chronic inflammation are the risk factors most likely to be responsible for the pathogenesis, in addition to hormonal changes due to pregnancy, lactation, and medical therapy [2]. Among the reviewed cases, (2/10) had been pregnant and/or lactating before, (1/10) had received an injectable contraceptive, and none had any past medical history. The reported case defies these assumptions as she has a negative medical, obstetric, and drug history and is too young to justify the effect of the aforementioned risk factors.

Clinically, the tumor most commonly presents as a painless, solitary mass in the upper quadrant of the breast, more commonly in the right breast [1]. Typical breast cancer findings, such as nipple discharge, skin discoloration and retraction, peau d’orange, and clinical axillary lymphadenopathy, are not common. Systemic symptoms at presentation are also uncommon [1,2]. Despite the lack of distinguishing clinical features, rapid growth and larger breast tumor size at diagnosis are noted to be more common in PBL than in other breast cancers [1]. About 12%-24% of presentations are incidental mammographic findings, and fewer are axillary masses [16]. In the presented and reviewed cases, 6 patients presented with a painless mass(s) or nodules, while the rest had other breast-related complaints. Bilateral presentation and systemic features were seen in (2/10) and (1/10) of the cases, respectively. B-symptoms were seen in (1/10) of the cases, as well as in the presented case.

The Breast Imaging-Reporting and Data System (BI-RADS) lexicon is utilized to depict malignancy potential. Mammography most commonly shows a hyperdense, lobular, solitary mass with indistinct margins but without calcifications, spicules, or anatomical distortions, and a BI-RADS category 3 [1,17]. Ultrasound mostly shows a solitary mass with indistinct and irregular margins that is hypoechoic with no posterior phenomena or axillary lymphadenopathy. Color Doppler, if available, shows hypervascularity [7,12,13,17]. MRI may show intense heterogeneous enhancement throughout with a type 3 kinetic curve [1,2,17]. The presented case deviates from these more common findings, as evident by the mixed echogenicity, similar to (2/10) other cases, with axillary and supraclavicular lymph node abnormalities on ultrasound, falling under category 4/4A, while the MRI showed a type 1 kinetic curve instead of 3. One other case provided a sonographic BI-RADS 5 category [12]. Imaging in (3/10), along with this case, revealed bilateral breast masses.

Fine needle aspiration followed by core needle, excisional, or incisional biopsy of the breast mass combined with IHC and FC are the main methods of diagnosis and subtype differentiation [1,2,16]. DLBCL is the most common diagnosis, while ALCL is the most common subtype of T-cell lineage [1,2]. Excisional or incisional biopsy in (3/10) and core needle biopsy of the breast in (5/10) of the cases were used, while the rest did not specify the method of obtaining the pathological specimen. IHC of the core needle biopsy in this case demonstrated the lymphoid origin of the infiltrating cells.

Staging is carried out using a PET/CT scan, with bone marrow biopsy and lumbar puncture, or CT with contrast of chest, abdomen, and pelvis if unavailable [2,7,11]. The most common stage at diagnosis is IE according to the Ann Arbor staging method [2,11]. The International Pediatric Non-Hodgkin Lymphoma Staging System is used by some as a staging method [7]. Among the reviewed cases, (3/10) had stage IIE, (1/10) stage III, (1/10) stage IV, while the rest did not provide a specific stage. Following CT with contrast imaging, the reported case received a stage IIE diagnosis, the same as the reviewed cases, but unlike what is found in large reviews.

First-line treatment in the majority was chemotherapy (CTx) with or without radiotherapy (RTx), the former being the main treatment method in aggressive tumors. The most common CTx regimen was CHOP+-R (7/10), including the current case.

CNS prophylaxis is becoming increasingly used due to the high risk of CNS relapse and occult disease, despite the lack of robust data supporting its use, while surgery is losing its role as a curative method [1,2]. Despite this, CNS prophylaxis was not given in any of the cases, as well as the presented case.

Different prognostic factors have been described, ranging from hematological and biochemical tests to performance status and age, with the International Prognostic Index for NHL remaining the most predictive of outcome. No specific method of follow-up is recommended, but CNS relapse, due to its poor outcome, has received the most attention [2,16]. Death was seen in (4/10) of the cases, in contrast to complete remission in (5/10). So far, the case has not shown treatment-related complications during the 4-month follow-up.

A limitation of this report is that no pathological specimen was obtained from the left breast masses to confirm their lymphoid origin.

## Conclusion

Young women are not exempt from PBL. CTx may offer good outcomes.

## Availability of data and materials

All data and materials are kept by the first and corresponding authors.

## Patient consent

Written informed consent was obtained from the patient for the publication of the present case report and any accompanying images and related investigations.
